# Brain topology improved spiking neural network for efficient reinforcement learning of continuous control

**DOI:** 10.3389/fnins.2024.1325062

**Published:** 2024-04-16

**Authors:** Yongjian Wang, Yansong Wang, Xinhe Zhang, Jiulin Du, Tielin Zhang, Bo Xu

**Affiliations:** ^1^Institute of Automation, Chinese Academy of Sciences, Beijing, China; ^2^School of Artificial Intelligence, University of Chinese Academy of Sciences, Beijing, China; ^3^Institute of Neuroscience, State Key Laboratory of Neuroscience, Center for Excellence in Brain Science and Intelligence Technology, Chinese Academy of Sciences, Shanghai, China; ^4^School of Future Technology, University of Chinese Academy of Sciences, Beijing, China; ^5^School of Life Science and Technology, ShanghaiTech University, Shanghai, China

**Keywords:** spiking neural network, brain topology, hierarchical clustering, reinforcement learning, neuromorphic computing

## Abstract

The brain topology highly reflects the complex cognitive functions of the biological brain after million-years of evolution. Learning from these biological topologies is a smarter and easier way to achieve brain-like intelligence with features of efficiency, robustness, and flexibility. Here we proposed a brain topology-improved spiking neural network (BT-SNN) for efficient reinforcement learning. First, hundreds of biological topologies are generated and selected as subsets of the Allen mouse brain topology with the help of the Tanimoto hierarchical clustering algorithm, which has been widely used in analyzing key features of the brain connectome. Second, a few biological constraints are used to filter out three key topology candidates, including but not limited to the proportion of node functions (e.g., sensation, memory, and motor types) and network sparsity. Third, the network topology is integrated with the hybrid numerical solver-improved leaky-integrated and fire neurons. Fourth, the algorithm is then tuned with an evolutionary algorithm named adaptive random search instead of backpropagation to guide synaptic modifications without affecting raw key features of the topology. Fifth, under the test of four animal-survival-like RL tasks (i.e., dynamic controlling in Mujoco), the BT-SNN can achieve higher scores than not only counterpart SNN using random topology but also some classical ANNs (i.e., long-short-term memory and multi-layer perception). This result indicates that the research effort of incorporating biological topology and evolutionary learning rules has much in store for the future.

## 1 Introduction

The mammalian brains, ranging from the simpler mouse brain to the more complex monkey and human brains, share some key functional circuits or brain regions to support different cognitive functions, including but not limited to sensation, memory, and decision-making. The brain network has been widely discussed in recent decades for its complexity (Luo, 2021). For example, the mouse brain network connectome at various scales has been largely examined, including the neuron-scale imaging of a cubic millimeter of mouse cortex (Yin et al., 2020), the mesoscale connectome of the entire mouse brain (Oh et al., 2014), and the macroscale network-motif topology analysis (Zhang et al., 2017). Many key topologies related to cognitive functions have been identified with the help of new optical or even electron microscopy, along with the well-designed experimental paradigms (Luo, 2021).

The mouse brain contains at least 213 brain regions, and the sparseness of the entire brain is < 36% (Oh et al., 2014), which makes it a good network reference to guide the design of spiking neural networks (SNNs) in especially neuromorphic computing manners (Maass, 1997). Until now, many key biological features have been incorporated into SNNs, including but not limited to neuronal heterogeneity, feed-forward or recurrent connections, and multiscale plasticity (Izhikevich, 2004; Zenke and Gerstner, 2017).

Different from artificial neural networks (ANNs), whereby single artificial backpropagation (BP) is used for network learning (Lillicrap et al., 2020), the learning algorithms in SNNs are various, such as plasticity-based algorithms [e.g., the spike-timing-dependent plasticity (Dan and Poo, 2004), short-term plasticity (Zhang et al., 2018), self-backpropagation (Zhang et al., 2021a)], gradient-based algorithms [e.g., reward propagation (Zhang et al., 2021b), surrogate gradient (Cramer et al., 2022)], and the evolutionary algorithms (Bäck and Schwefel, 1993).

However, there is a serious conflict between biological topology and corresponding learning rules since a predefined topology will usually be revised or destroyed by gradient or plasticity-based algorithms (Bellec et al., 2020). Here we run further by considering some evolutionary algorithms, which have also been verified efficient in tuning SNNs for their simplicity and efficiency, and what's most important, resolving this conflict problem by selectively pruning some trivial branches in network topology during learning.

In this paper, the main goal is to incorporate some subsets of brain topology (BT) into SNNs, and then train them using an evolutionary algorithm during reinforcement learning (RL) tasks. The detailed process and contribution of this paper can be concluded in the following parts:

- Some important subsets of network topology are filtered out from the source brain topology by considering some biological constraints. As a result, three key BTs have been generated from the mesoscale connectome of Allen mouse brain atlas (Oh et al., 2014) by the Tanimoto hierarchical clustering algorithm. Different types of BTs are further analyzed by the distribution of the three-node network motif to answer why the topology might work from the perspective of intuitive biological analysis.
- The BT-improved SNNs (BT-SNNs) are designed by incorporating different types of BTs and SNNs using numerical solver-improved leaky integrate-and-fire neurons, whereby an evolutionary-type learning algorithm is used to efficiently guide the synaptic modification without affecting key network topology.
- Four benchmark RL tasks in OpenAI Mujoco environment (Brockman et al., 2016), also with some key features of animal survival, are used to test the performance of the proposed algorithm, including MountainCar-v2 (a car learns to stop at a mountain), Half-Cheetah-v2 (a dog that learns to run), Humanoid-v2 (a human that learns to run), and HumanoidStandup-v2 (a human that learns to stand up). The BT-SNNs have reached a higher average reward than their counterpart algorithms, including SNNs using random topologies and classical ANNs, such as long-short-term memory (LSTM) and multi-layer perception (MLP).

## 2 Related works

Borrowing key topology knowledge from different animal brains is challenging, caused by raw data analysis and topology-informed computation. For the network topology, a copy-and-paste approach, i.e., copying the structural synaptic connectivity map of a mammalian brain and pasting it to a three-dimensional network in solid-state memories of neuromorphic engineering, has been proposed with the spirit of reverse-engineering the brain (Ham et al., 2021). Some distilling algorithms try to make an abstraction of a teacher network to a much smaller student network, but with less computational cost and comparable performance (Han et al., 2015; Hinton et al., 2015). The biological topology-focused algorithm by using a sub-graph sparse network to replace a previous global dense one, named as lottery ticket hypothesis, has been proposed to achieve comparable or even higher performance (Frankle and Carbin, 2018). Some researchers believe that the network topology and synaptic weights are two independent dimensions. Hence, they focus more on learning synaptic weights and leave the topology fixed with feed-forward, recurrent, or some scale of sparseness topology. A new study focuses on these two aspects both by learning weights and topology simultaneously toward a much more efficient algorithm (Han et al., 2015). Similar to it, a biological network using *C. elegans* topology has also been proposed to achieve higher scores in RL paradigms than those using random topology, which to some extent, indicates the efficiency of the biological topology in network learning (Hasani et al., 2020).

SNNs frequently underperform relative to ANNs in handling complex tasks (Deng et al., 2020). There are studies that apply deep learning, gradient descent, and backpropagation to biologically reasonable SNNs (Eshraghian et al., 2023). There are also studies using neural pruning methods to implement adaptive sparse learning SNN (Li et al., 2024). Some studies using knowledge distillation and connection pruning methods to dynamically optimize synaptic connections in SNN (Xu et al., 2023).

Some studies have instantiated Biological Neuronal Networks (BNNs) into Recurrent Neural Networks (RNNs) for network structure exploration (Goulas et al., 2021). Some people also combine the feature learning ability of CNN with the cognitive ability of SNN to improve the robustness of SNN (Xu et al., 2018), and some other works have emulated the brain's synaptic connections and dynamic behaviors through Nanowire Networks (NWNs) to facilitate learning and memory functions (Loeffler et al., 2023).

For the learning algorithms under RL tasks, a multiscale dynamic coding algorithm has been proposed to improve an SNN on OpenAI Mujoco tasks (Zhang et al., 2022). Besides, a traditional continuous-time differential learning algorithm has been proposed for RL tasks containing continuous dynamics (Doya, 2000). A hybrid learning framework, incorporating SNNs for energy-efficient mapless navigation, has been proposed and applied on the neuromorphic hardware (Tang et al., 2020).

However, most of these proposed algorithms overlook the importance of network topology in learning, especially the exploration of inter-cluster topological relationships within brain regions, and neglect some key features by following gradient-based or plasticity-based algorithms. The further incorporation of network topology, especially those related to cognitive functions of sensation, motor, and reward learning, can exhibit more power on animal-survival-like RL tasks. It is becoming an important consensus that the topology is at least as important as synaptic weights to the network performance. Here we employ a hierarchical clustering algorithm to generate some network topology from the Allen mouse brain atlas first and then incorporate a standard evolutionary algorithm to guide the synaptic modification without using traditional gradient and plasticity-based rules.

## 3 Methods

### 3.1 The raw brain topology in Allen mouse brain atlas

Analyzing a set of biological topologies is usually the first step to support the following network-topology simulation in neural networks. Here we select the mesoscale Allen mouse brain atlas provided by the Allen Institute for Brain Science (Oh et al., 2014). It contains publicly available resources on brain region morphology (e.g., the common coordinate framework, CCF) and mesoscale network topology at sub-brain region scale which covers bidirectional topology in 213 brain regions.

A 3D model containing at least 213 brain regions is first constructed based on the mouse brain CCF for visualization, analysis, and functional simulation (see Section 4 for more details). The 213 brain regions are separated into three subgroups: the sensation group, including but not limited to the primary somatosensory area, primary visual area, primary auditory area, and accessory olfactory bulb; the motor group, including but not limited to the primary motor area, dentate nucleus, and motor nucleus of trigeminal; the left brain regions except the previously mentioned two groups but related to some key cognitive functions, including but not limited to the hippocampus for memory, basal ganglia for reward learning.

**Figure 1:**
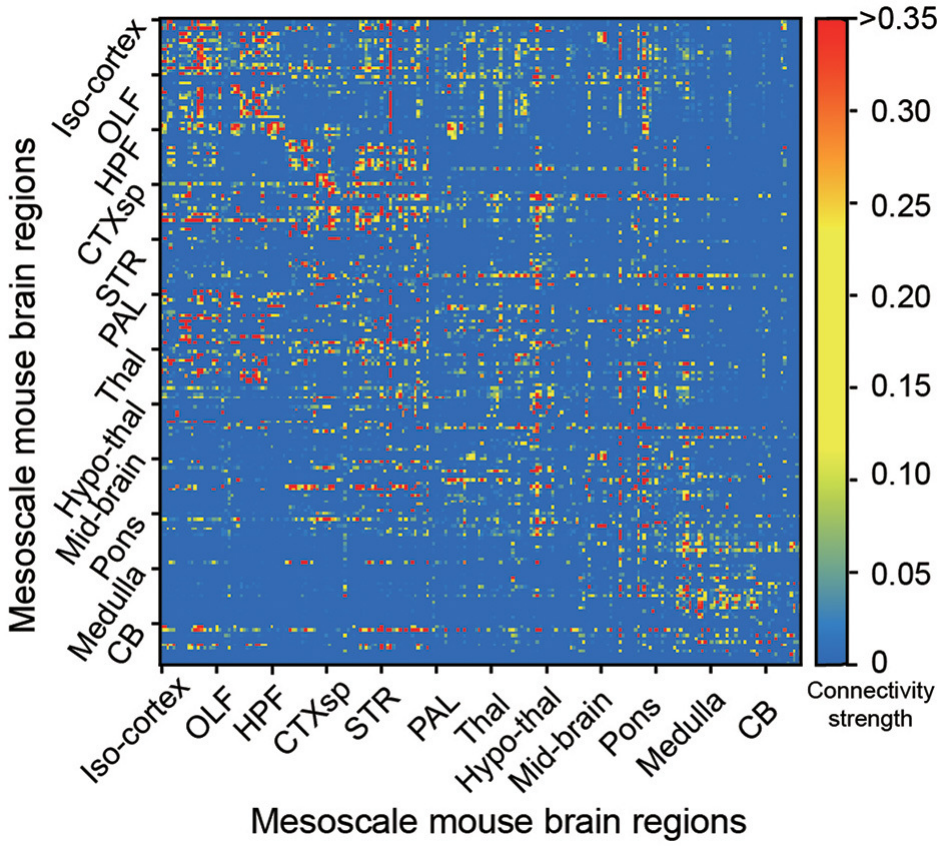
The mesoscale connectome of the Allen mouse brain atlas in 213 brain regions. Each dot represents the connectivity strength, with a color bar aside from the figure to represent the strong (red) or weak (blue) strength, from each source (y-axis) to target (x-axis) brain regions in the whole mouse brain (Oh et al., 2014).

The bidirectional connectivity of the whole Allen mouse brain is shown in Figure 1, containing the mapping connectivity from a source brain region to a target region in the total 213 brain regions (Oh et al., 2014). It is easier to find that the connectivity matrix is much sparser, which is considered the key feature of biological structures compared to those in recurrent neural networks.

### 3.2 The Tanimoto hierarchical clustering

The connectivity matrix of the mouse brain atlas (213 × 213 size) is clustered into sub-clusters for an easier simulation. The Tanimoto clustering algorithm is selected as the main method to group all connections (Ahn et al., 2010; Kalinka and Tomancak, 2011), which could be concluded as the following Equation 1, where *S*(*e*_*i, k*_, *e*_*j, k*_) represents the similarity between links *e*_*i, k*_ and *e*_*j, k*_ that share a node *k*:


(1) $$\begin{array}{l} S\left(e_{i,k},e_{j,k}\right)=\frac{a_{i}\cdot a_{j}}{|a_{i}{|}^{2}+|a_{j}{|}^{2}-a_{i}\cdot a_{j}}, \end{array}$$

where the vector *a*_*i*_ = (Ã_*i*1_, ..., Ã_*iN*_) describes the connectivity strength between the node *i* and its first-order neighborhoods, and the Ã_*i, j*_ is set as the following Equation 2:


(2) $$\begin{array}{l} {Ã}_{i,j}=\frac{1}{k_{i}}\underset{i^{\prime }\in n\left(i\right)}{\sum }w_{i,i^{\prime }}\delta_{i,j}+w_{i,j}, \end{array}$$

where *w*_*i, j*_ is the connectivity strength for edge *e*_*i, j*_, *n*(*i*) is a neighborhood set defined as {*j*|*w*_*i, j*_ > 0}, *k*_*i*_ = |*n*(*i*)|, and δ_*i, j*_ = 1 when *i* = *j* or else δ_*i, j*_ = 0. Then the dendrogram can be cut at a large partition density height to get link and node clusters. The detailed Tanimoto hierarchical clustering algorithm can be found at Algorithm 1.

**Algorithm 1:** Tanimoto hierarchical clustering.

Input: All connections and Tanimoto coefficient *S*;
Assign each connection to its own cluster;
Sort *S* from large to small as $S_{\left(L_{1},L_{1^{\prime }}\right)}$, ..., $S_{\left(L_{N},L_{N^{\prime }}\right)}$;
i = the number of clusters, j = 1;
**for** i > 1 and j < N+1 **do**
Merge the clusters which contain connection *L*_*j*_ and $L_{j^{\prime }}$;
Encode $S_{\left(L_{j},L_{j^{\prime }}\right)}$ as the height;
Store the process in the dendrogram;
j = j + 1, i = the number of clusters;
**end for**Output: The dendrogram.

After the Tanimoto clustering, a community of sub-connectivity matrices in 213 brain regions can be hierarchically separated at a desired partition density. The partition density *D* can be calculated as the following Equation 3.


(3) $$\begin{array}{l} D=\frac{2}{M}\underset{i}{\sum }\frac{m_{i}+1-n_{i}}{\left(n_{i}-2\right)\left(n_{i}-1\right)}, \end{array}$$

where *m*_*i*_ is defined as the number of connections giving a specific cluster *i*; *n*_*i*_ is defined as the number of nodes in the same cluster *i*, and *M* is the number of connections for the whole network which contains all clusters. The *D* indicates the density of connections, with its value adjusted relative to the theoretical maximum and minimum connection scenarios within the network. This adjustment allows for a standardized comparison of connection densities across different network configurations.

The community connectedness of cluster *i*, as defined by Equation 4, quantifies the degree of connection between cluster *i* and other clusters, reflecting a comparison of external connections to other clusters relative to internal connections within the cluster itself. For clusters containing a large number of brain regions, this value tends to be lower (e.g., 10–20), indicating a higher proportion of internal connections. If the value is too low (< 10), there may be artifacts that interfere with the value of statistical research.


(4) $$\begin{array}{l} C_{i}=\frac{n_{i}\left(n_{i}-1\right)e_{b}\left(i\right)}{2e_{w}\left(i\right)n_{i}\hat{d}}, \end{array}$$

where *n*_*i*_ is defined as the number of nodes within the cluster *i*; *e*_*b*_(*i*) is defined as the number of connections between cluster *i* and its neighborhood clusters; *e*_*w*_(*i*) is defined as the number of connections within the cluster *i*. $\hat{d}$ is defined as the whole-network average degree.

### 3.3 The brain topology

**Figure 2:**
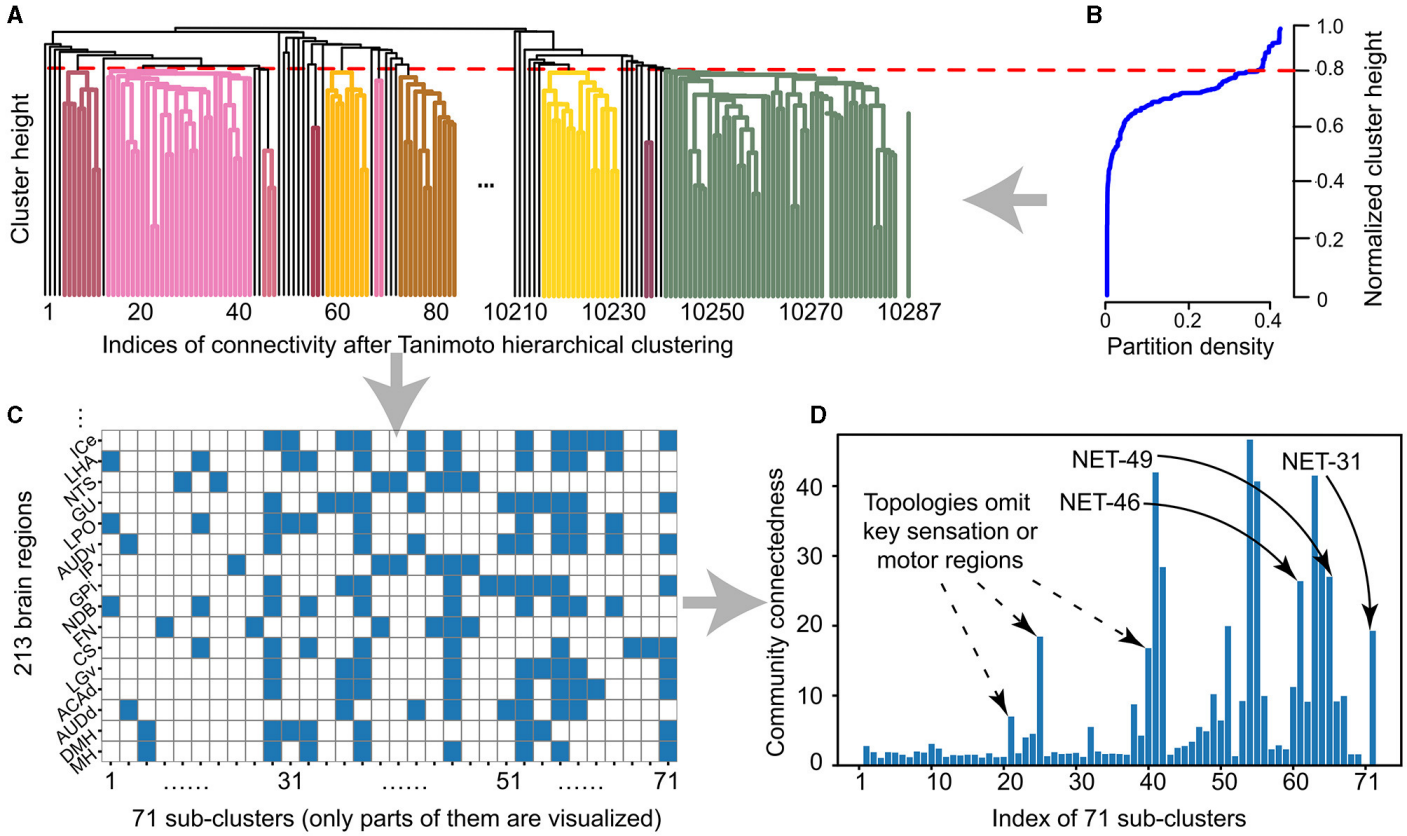
The sub-clusters from 213 brain regions after hierarchically Tanimoto clustering and sparseness constrain. **(A)** The 71 clusters are generated by the hierarchical Tanimoto clustering algorithm from 213 brain regions and 10,287 connections. **(B)** The relationship between clustering height and partition density, as those described in the Equation 3. Here we set the clustering height as 0.8 after considering both the sparseness and size of each sub-cluster and then we get 71 sub-clusters accordingly. **(C)** In 71 sub-clusters, 213 brain regions (color squares) sparsely participate in each topology indexed from 1 to 71. **(D)** The relationship between sub-clusters and community sparseness. Three selected sub-clusters with the indices of 71, 61, and 65 (labeled as the start point) with the number of brain regions of 31, 46, and 49, respectively, respectively. These three types of networks are named as BTs for next-step learning.

The brain-region clusters are generated from the 213 brain regions of the Allen mouse brain atlas by the Tanimoto clustering algorithms first, and then biological experts make a selection by considering some biological constraints. The 71 sub-clusters after clustering are concentrated in three intervals, < 10 nodes, 30 to 60 nodes, and greater than 100 nodes. Taking into account the clustering principle and the artifacts present in the experiment, the interval of 30 to 60 nodes is the most preferred for brain topology experiments. Considering the subsequent tasks such as Mujoco, the key clusters we study need to have sensory, memory and motor functions. The detailed procedure of brain-region clustering contains five steps:

- The Tanimoto clustering algorithm is used to make a hierarchical clustering of these 213 brain regions. Different brain regions can be generated at different clustering height levels, as shown in Figure 2A.
- The selection of clustering height is inducted by biological experts. A smaller or bigger clustering height will cause the partition density to be too small or too big, representing allocating all brain regions into the same cluster or an independently different one, respectively. Then the clustering height (sparseness) is set as 0.8 and get 71 clusters, as shown in Figure 2B and Equation 3.
- The proper density is verified by visualizing the participation of each brain region in each cluster generated in the previous step, as shown in Figure 2C.
- As can be seen from Figure 2C, according to the sparsity designed in Figure 2B, the design of our 71 cluster factor Tanimoto clustering method shows connectivity at different scales. There are two conditions for sub-clusters to be selected for further processing: first, the connectivity is in the appropriate range, that is, the community connectedness is 10–40 in Figure 2D; second, it is biologically reasonable, that is, the cluster includes brain areas with sensory, memory, and motor functions.
- Some key clusters (i.e., three ones after analysis) with a different number of brain regions (i.e., the cluster with the index of 31, 46, and 49) are generated and named the NET-31, NET-46, and NET-49.

During these five steps, the brain-region clusters can be automatically generated as candidate clusters, which are efficient without a time-consuming manual summary, which is important for the efficient network topology generation at the whole mouse-brain scale.

### 3.4 The biologically-plausible SNNs

Both the leaky-integration neuron (LI) (Hasani et al., 2020) and leaky integrate-and-fire (LIF) neuron (Liu and Wang, 2001) with excitatory and inhibitory types are used as the basic neuron model for the next-step simulation of SNNs at the network scale. The design of the LI model is represented as the following Equation 5.


(5) $$\{\begin{array}{l} {\dot{V}}_{i,t}=\left[I_{L}+\sum {\hat{I}}_{C,t}+\sum I_{C,t}\right]/C_{m}\\ I_{L,t}=\omega_{L}\left(E_{L}-V_{post,t}\right)\\ I_{C,t}=\omega_{C}\left(E_{C}-V_{post,t}\right)g_{t}\\ g_{t}=1/[1+exp(-\sigma (V_{pre,t}-\mu ))] \end{array},$$

where *C*_*m*_ is the membrane capacitance of the neuron, *I*_*C, t*_ and *I*_*L, t*_ are the input currents of the chemical and leakage channels, respectively. *E*_*C*_ and *E*_*L*_ are the corresponding reversal potentials. *V*_*post, t*_ and *V*_*pre, t*_ are the membrane potentials of post-synapses and pre-synapses, respectively. *g*_*t*_ is the dynamic conductance of the membrane, defining whether a synapse is excitatory or inhibitory by *E*_*C*_. ω_*C*_ and ω_*L*_ are the conductance in chemical and leakage channels, respectively.

The LI neuron can realize the adaptive calculation of the ordinary differential equation (ODE) and has a strong ability to model the time series reaching a goal at any time step. Besides LI neurons which play key roles in the inner dynamics in the hidden layers of networks, we also introduce the sensory and motor neurons in the input and output layers, respectively, during the interaction with the environment.


(6) $$\{\begin{array}{l} {\dot{V}}_{i,t}=\left(I_{L}+\sum {\hat{I}}_{C,t}+\sum I_{C,t}\right)\left(1-S_{i,t}\right)/C_{m}\\ S_{i,t}=V_{i,t}>V_{th} \end{array}$$

A hybrid numerical solver (Press et al., 2007) is used and combines with explicit Euler's discretization (Lechner et al., 2019), similar to that in Hasani et al. (2020), where a fixed-step solver is used to calculate ODE, and at each time step Δ_*t*_, our approach complexity is around *O*(|*N*_*n*_|+|*N*_*s*_|), where *N*_*n*_ is the number of neurons, and the *N*_*s*_ is the number of synapses, as shown in Equation 6.

After the membrane potential *V*_*i, t*_ reaches the firing threshold *V*_*th*_, the spiking flag *S*_*i, t*_ is set as true, which will reset the update of the membrane potential *V*_*i, t*_ by multiplying 1−*S*_*i, t*_, with the spirit of biological leaky integrate-and-fire.

### 3.5 BT improved SNN

Biological experts group the 213 brain regions in the Allen mouse brain atlas into three subgroups. The first group is the input layer containing the sensation-related brain regions, e.g., the primary somatosensory and visual areas. The second group is the hidden layer containing the cognitive-function-related brain regions, e.g., the hippocampus and basal ganglia. The third group is the output layer containing the motor-related brain regions, e.g., the primary motor area and trigeminal motor nucleus. We also annotate the biological functions of the brain regions at each level of the clusters of interest (see Section 4 for more details), which directly link the biological regions to network layers.

Besides the topology with 213 brain regions (which can be considered the whole brain region, NET-213), different types of network topology with different numbers of brain regions are selected by biological experts for the next-step simulation. Using the configuration of the 0.8 sparseness during the hierarchical clustering level (Figure 2B), we select brain regions with the index of NET-31, NET-46, and NET-49 in all 71 sub-clusters (Figure 2C), where each number represents the number of brain regions in the selected topology. These clusters all cover sensation, cognitive function, and motor brain regions without omitting the key transfer region in a network (Figure 2D). The detailed brain regions in NET-46 will be further introduced in Section 4.

### 3.6 The evolutionary-based learning algorithm

The SNN with biological topology (i.e., connected to each node with the biological network NET-213, NET-31, NET-46, and NET-49) can be tuned by many learning algorithms. Here we select the evolutionary-based algorithms for their topology-friendly advantages, i.e., the adaptive random search algorithm (ARS) (Hasani et al., 2020). We find it can also get around some serious problems in recurrent neural networks during reinforcement learning, including but not limited to gradient scaling problems and long-term dependence problems (Mania et al., 2018).

In this paper, we optimize the ARS algorithm and use it in RL tasks, whereby the agent learns to make decisions after observing the current state in an environment and then receives a timely or delayed reward. The fitness function is designed to collect these rewards and guide the direction of the random search. At the beginning of network learning, the agent makes random decisions for exploration, and a good decision for a lower fitness function will be kept by saving the current parameters and focusing more on the exploitation. The search-based algorithm ARS can train a network by repeating two training strategies until convergence. First, expected values are obtained by perturbation network parameters. Then the adaptive search algorithm calculates the distance between expected values and fitness function and uses it further to guide the search space for a smaller distance. Objectively, the ARS algorithm requires a certain amount of effort to identify and select potentially useful network structures, and the network learning convergence using ARS is slower than the standard gradient-based algorithms, where the desired gradient is calculated by re-sampling the dataset in a memory buffer. However, the memory buffer makes at least two serious problems: (1) the extremely high storage space; (2) the re-sampling of samples collected from the exploration is inefficient. Hence, the ARS can save computational costs without considering the storage space and re-sampling than the standard gradient-based algorithms, which indicates it is more suitable for online and neuromorphic computation.

### 3.7 The analysis of BTs using network motif

The NET-31, NET-46, and NET-49 contain many brain regions (with input, hidden, and output areas) and sparse connections. Here we use 2D and 3D visualization methods to highlight the main difference between these three network topologies.

**Figure 3:**
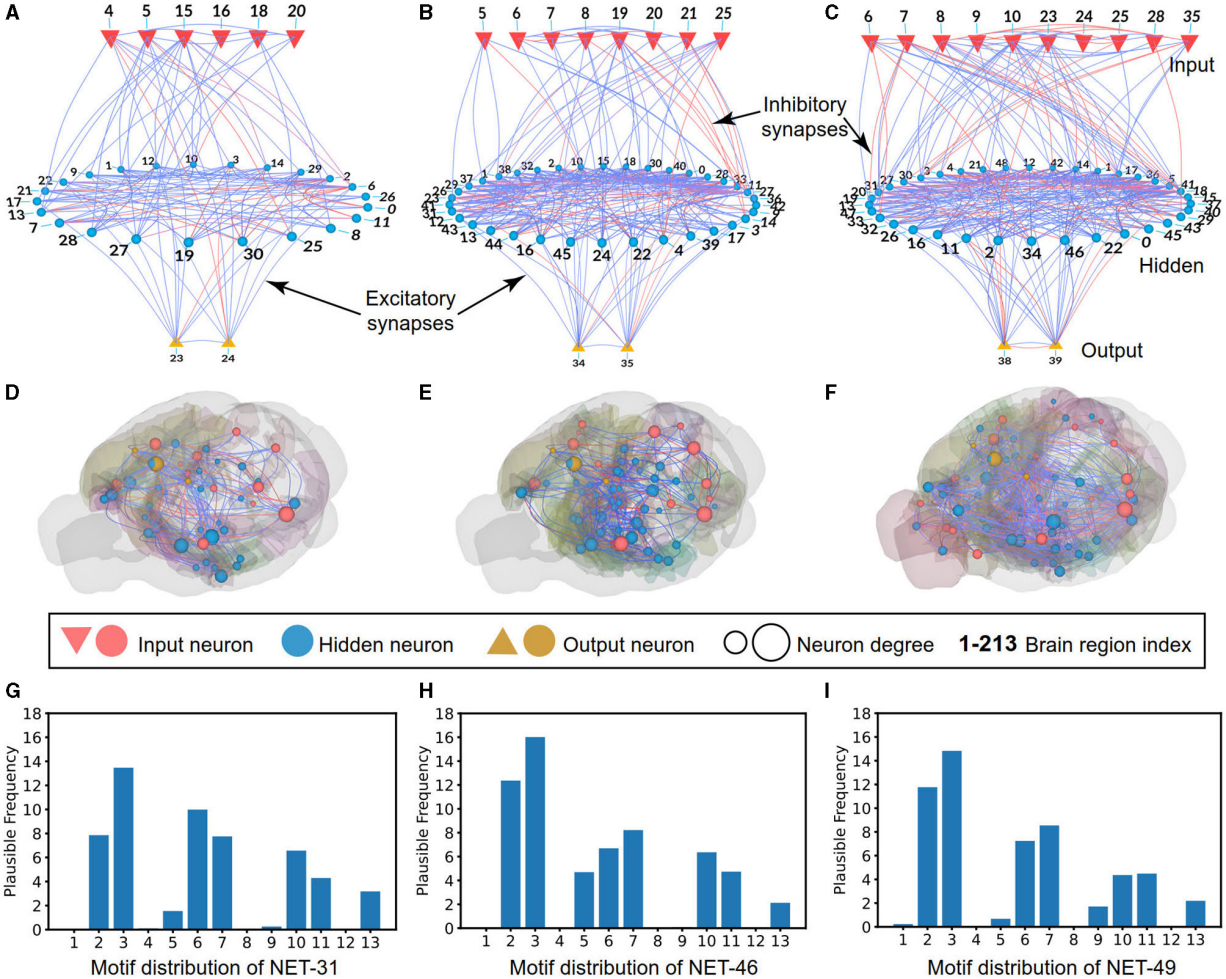
Schematic diagrams of BTs with visualization. **(A–C)** The 2D visualization describes the input (red inverted triangles), hidden (blue circles), and output (regular yellow triangles) brain regions in NET-31, NET-46, and NET-49, respectively. The blue and red lines represent excitatory and inhibitory connections between regions, respectively. **(D–F)** Same as those in **(A–C)** but with 3D visualization in a mouse brain, whereby sensory regions (red sphere), memory regions (and other cognitive function regions, blue sphere), and motor regions (yellow sphere) with sparse connections are visualized. **(G–I)** Motif distribution of NET-31, NET-46, and NET-49, respectively. The horizontal axis represents 13 different types of motifs, and the vertical axis represents the credible frequency, defined as the frequency of the motif multiplying the confidence value (with 1 deleting *P*-value).

For the 2D visualization, as shown in Figure 3A, the NET-31 contains six input regions, 23 hidden regions, and two motor regions. A total of 450 connections are plotted, containing 361 excitability and 89 inhibitory connections. As shown in Figure 3B, the NET-46 contains eight input regions, 36 hidden regions, and two motor regions. A total of 802 connections are plotted, containing 575 excitability and 227 inhibitory connections. As shown in Figure 3C, the NET-49 contains ten input regions, 37 hidden regions, and two motor regions. A total of 904 connections are plotted, containing 713 excitability connections and 191 inhibitory connections. The definition of the ratio of excitatory neurons is 70%, the same as that found in the brain cortex (Wildenberg et al., 2021).

For the 3D visualization of three networks (i.e., NET-31, NET-46, and NET-49), the connections of different brain regions from input, hidden, and motor areas are given under a background of the mouse-brain CCF. With the help of biological experts, the input regions belong to the occipital lobe, the output regions belong to the parietal lobe, and the hidden regions are everywhere in the brain for the complex information processing, consistent with the biological functions, as shown in Figures 3D–F. For ease of visualization, the connections with connectivity strength lower than 0.05 in three networks are omitted. For example, only 207 excitatory and 60 inhibitory connections are visualized in NET-31.

The 3-node network motif (Milo et al., 2010) has been widely used to analyze the dynamic properties (Prill et al., 2005) and biological network features (Sporns and Kotter, 2004). Here we also use the 3-node network motif to analyze the connection distribution feature of the NET-31, NET-46, and NET-49. As shown in Figure 3, we use the “credible frequency” (the product of the occurrence frequency and 1−*P*) instead of the pure frequency to avoid the influence of some random features. Here *P* is the *P*-value of each motif in the selected network compared to the 1,000 randomly generated networks of the same size. Each generated network is sampled from a uniformly random distribution. The smaller *P*-value, the less likely a random network will have the same network features as a biological one.

In all calculated network motifs, we want to highlight the motif-5 distribution (a type of cross-layer connection). The motif distributions for the three topologies share some common features, such as the motif-1, 2, 6, 7, 10, 11, and 13 are relatively higher than other motifs. The motif-5 and motif-9 are the main two differences that might be the main differences of functional circuits in these three topologies. Further analysis will be given in the performance comparison of these three networks.

## 4 Experiments

### 4.1 Introduction of tasks and implement details

**Figure 4:**
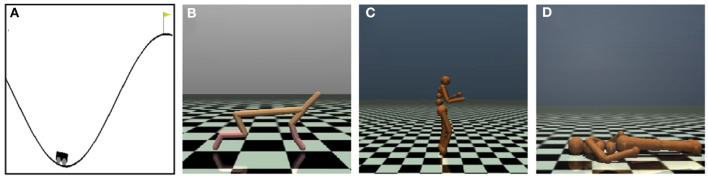
Schematic diagram depicts four OpenAI Mujoco tasks for continuous controlling. **(A)** The MountainCar-v2 task drives to the top mountain as fast as possible. **(B)** The Half-Cheetah-v2 task makes a 2D cheetah robot run as fast as possible. **(C)** The Humanoid-v2 task makes a three-dimensional bipedal robot walk forward as fast as possible without falling over. **(D)** The HumanoidStandup-v2 task makes a three-dimensional bipedal robot stand up as fast as possible.

Four OpenAI gym games (Mujoco) were used to test the algorithms' performance, as shown in Figure 4. We select these Mujoco tasks instead of Atair 2000 games for their more dynamic features, especially animal-survival-like RL (Figures 4B–D).

In order to ensure the reproducibility of the proposed algorithms, we repeated each RL experiment ten times with different network initializations given different random seeds. Each RL task took 300 k (1k = 1,000) steps for learning and was evaluated every 10k step. At each evaluation time, we reported the average reward of over 10 episodes without giving any exploration noise, and each episode lasted for a maximum of 1 k execution steps. The MountainCar-v2, Half-Cheetah-v2, Humanoid-v2, and HumanoidStandup-v2 tasks are with state-action dimensions of [2, 1], [17, 6], [376, 17], and [376, 17], respectively. All these RL-related configurations are similar to those in the paper (Hasani et al., 2020), where a simpler network architecture borrowed from *C. elegans* is used.

We compared our algorithms to the benchmark LSTM and MLP networks. The experiments were built upon the open-source codebase from OpenAI Spinning Up.^1^ The related algorithms, including NET-31, NET-46, NET-49, and Net-Rand, were all trained under the same standard ARS algorithm. We evaluated these algorithms on the four continuous control tasks under the same experimental configurations and compared their performance for further analysis. Unless for special statements, most algorithms use the same set of parameters.

### 4.2 Performance comparison of SNNs using different BTs

**Figure 5:**
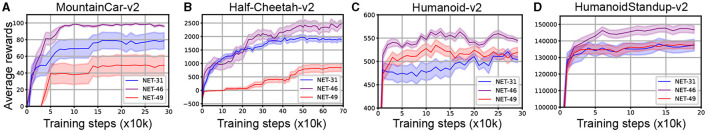
Performance comparisons of SNNs employing NET-31, NET-46, and NET-49 across four continuous control RL tasks: **(A)** MountainCar-v2, **(B)** Half-Cheetah-v2, **(C)** Humanoid-v2, and **(D)** HumanoidStandup-v2. The x-axis measures training steps (x10k), and the y-axis displays average rewards. Shaded regions indicate standard deviation. In task A, achieving the mountain top is marked by a score of 100.

The performance of SNNs using three types of topology on four reinforcement learning tasks is shown in Figure 5. From the statistical results, the performance of SNNs using NET-46 is better than those using NET-31 and NET-49, representing NET-46 could be the main best-topology candidate in the next experiments for comparing its performance with random networks and other state-of-the-art algorithms.

For the different distribution of network motifs in three BTs, it is obvious that motif-5 occupied a higher proportion in NET-46 than NET-31 and NET-49 (see Figures 3G–I for more details). It is impressive that the motif-5 contains a more cross-layer connection, making us speculate that the proper proportion of cross-layer connections plays a significant role in RL tasks. The motif-9 is another main difference between these three topologies. However, the influence of motif-9 is opposite to motif-5, where networks using more motif-9 exhibited poorer performance than other control algorithms.

### 4.3 SNNs using NET-46 V.S. SNNs using random topology

The SNN using NET-46 exhibit a superior performance than SNNs using NET-31 and NET-49. However, we cannot claim the NET-46 is the best BT candidate without comparing it to an objective benchmark as the baseline. Hence, we select two types of benchmarks for verification: (1) the bottom baseline is defined by the SNN using a random network, given the name of NET-Rand; (2) ANNs define the top baseline using MLP or LSTM, which will be introduced extensively in the next section.

**Figure 6:**
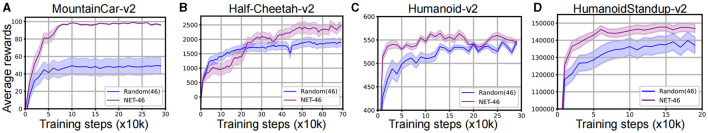
Comparative analysis of SNNs using NET-46 and baseline NET-Rand on the four continuous control RL tasks: **(A)** MountainCar-v2, **(B)** Half-Cheetah-v2, **(C)** Humanoid-v2, and **(D)** HumanoidStandup-v2. NET-46 outperforms NET-Rand, as shown by the higher average rewards. The horizontal axis indicates training steps (x10k), and the vertical axis represents average rewards.

For the bottom baseline, we conducted a topology with the same number of brain regions and connections to the NET-46. The ratio of excitatory to inhibitory connections was 0.7 to 0.3. The SNN using NET-Rand was trained on the four RL tasks, and the inference performance comparison of it and NET-46 was shown in Figure 6 and Table 1. The experimental results showed that the performance of SNNs using the NET-rand was much lower than those using NET-46, which to some extent, indicated that the NET-46 contains some key topology advantage for the efficient RL. The performance on the MountainCar-V2 task was higher than other tasks, which the less complexity might cause.

**Table 1:** The performance comparisons in Mujoco RL.

**Tasks**	**Architectures**	**Rules**	**RL Scores**	**Network sparsity**
MountainCar-v2	LSTM^a^	BPTT	98.98 ± 0.59	0%
	MLP^b^	PPO	95.5 ± 1.5	0%
	Random	Search	49.59 ± 49.59	61%
	**NET-46 (Ours)**	**Search**	**99.14** **±0.12**	**61%**
HalfCheetah-v2	LSTM^a^	BPTT	1009.51 ± 641.95	0%
	MLP^b^	PPO	1,601.05 ± 506.50	0%
	Random	Search	1,917.40 ± 819.39	61%
	**NET-46 (Ours)**	**Search**	**2,468.18** **±962.36**	**61%**
Humanoid-v2	LSTM^a^	BPTT	537.19 ± 33.54	0%
	MLP^b^	PPO	537.36 ± 39.73	0%
	Random	Search	545.65 ± 32.79	61%
	**NET-46 (Ours)**	**Search**	**564.13** **±37.65**	**61%**
HumanoidStandup-v2	LSTM^a^	BPTT	142255 ± 7502	0%
	MLP^b^	PPO	140,780 ± 9,676	0%
	Random	Search	139,863 ± 24,538	61%
	**NET-46 (Ours)**	**Search**	**147,697** **±11,931**	**61%**

^a^Citation for LSTM (Hochreiter and Schmidhuber, 1997).^b^Citation for MLP (Schulman et al., 2017). The metrics in bold are the corresponding metrics for our model, just for highlighting.

Furthermore, the enhanced performance of NET-46 over NET-Rand in our experiments can be attributed to its biologically-informed structural properties, such as optimized connectivity patterns and modularity, which are absent in randomly generated networks. The SNN using NET-Rand could also be convergence but only with lower average rewards. Hence, now we can answer the hypothesis from the computation perspective that the evolutionary neural networks have stored some key prior knowledge in brain topology, which further contributes to the next-step network learning. The top baseline is then tested and showed in the following section.

### 4.4 SNN using NET-46 V.S. classical ANNs

**Figure 7:**
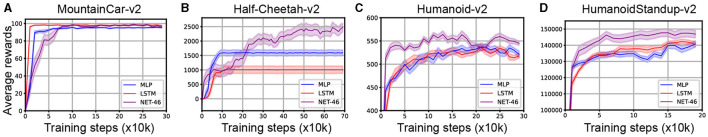
Performance comparison of the SNN with NET-46 against the ANNs using LSTM and MLP in four RL tasks: **(A)** MountainCar-v2, **(B)** Half-Cheetah-v2, **(C)** Humanoid-v2, and **(D)** HumanoidStandup-v2. NET-46 demonstrates superiority over MLP and LSTM. Training steps and average rewards are depicted on the x and y axes, respectively.

We selected the MLP and LTSM and tested their performance on the four Mujoco continuous control RL tasks. The experimental results are shown in Figure 7. For the MountainCar-v2 task, our algorithm (i.e., SNN using NET-46) reached a comparable performance (99.14 ± 0.12) to the other two benchmark algorithms, including LSTM (98.98 ± 0.59, *n* = 10, *P* = 0.94) and MLP (95.5 ± 1.5, *n* = 10, *P* < 0.01). For the other three relatively more complex tasks, our algorithm performed much better and reached a higher performance than LSTM with [*P* value = 0.01, *P* = 0.12, and *P* = 0.04] and MLP with [*P* = 0.01, *P* = 0.19, and *P* = 0.15] for Half-cheetah-v2, Humanoid-v2, and HumanoidStandup-v2 RL tasks, respectively. See Table 1 for more details. It should be noted that although the brain-like topology algorithm represented by NET-46 in this article has better computational performance than MLP and LTSM algorithms, the SNN still has some room for further improvement in terms of computational cost.

## 5 Conclusion

Incorporating biological topology into SNNs can provide insights into the structural organization of neural networks. This study utilized the mesoscale connectome data from the Allen mouse brain atlas, involving 213 mouse brain regions, to explore how specific topological clusters (i.e., NET-31, NET-46, and NET-49) can be clustered, analyzed, filtered, and incorporated into SNNs. The focus was on examining the structural compatibility of these clusters with SNN architectures, aiming to understand their potential influence on network performance.

These three clusters' excitatory-inhibitory connection types and sparseness are consistent with the biological ones, including sensory, hidden (for memory), and motor brain regions. The three BTs exhibited different performances during RL, and the NET-46 outperformed NET-31, NET-49, and the random network (NET-Rand). The detailed brain regions in NET-46 contain more auditory brain regions, more hidden brain regions for memory and multi-sensory integration, and more global neuromodulatory pathways, such as 5-HT projections from the CLI region to the nucleus and thalamus.

The experimental results showed that the mouse brain-like topology could improve SNNs from the perspective of accumulated rewards and network sparsity more than some ANNs, including the LSTM and MLP. We think more biological network-scale principles can further be incorporated into SNNs, and this integration of neuroscience and artificial intelligence has much in store for the future.

## Data availability statement

The datasets presented in this study can be found in online repositories. The names of the repository/repositories and accession number(s) can be found in the article/supplementary material. The mouse brain topology dataset can be downloaded from http://connectivity.brain-map.org. The source code of this paper can be found at https://github.com/thomasaimondy/BT-SNN.

## Author contributions

YoW: Formal analysis, Methodology, Resources, Software, Validation, Writing – original draft, Writing – review & editing. YaW: Data curation, Formal analysis, Methodology, Validation, Visualization, Writing – original draft. XZ: Formal analysis, Methodology, Supervision, Writing – review & editing. JD: Supervision, Funding acquisition, Writing – review & editing. TZ: Formal analysis, Methodology, Resources, Writing – original draft, Writing – review & editing. BX: Supervision, Funding acquisition, Writing – review & editing.

## Footnotes

^1^ https://github.com/openai/spinningup
